# Associations of early childhood caries with salivary beta defensin-3 and childhood anemia: a case–control study

**DOI:** 10.1186/s12903-021-01810-x

**Published:** 2021-09-14

**Authors:** Sanam Faheem, Shahida Maqsood, Arshad Hasan, Fouzia Imtiaz, Faheem Shaikh, Waqas Ahmed Farooqui

**Affiliations:** 1 Department of Oral Biology, Dow Dental College, Dow University of Health Sciences, Karachi, Pakistan; 2 Department of Operative Dentistry, Dow Dental College, Dow University of Health Sciences, Karachi, Pakistan; 3 Department of Biochemistry Dow Medical College, Dow University of Health Sciences, Karachi, Pakistan; 4 London Dental Clinics & Dental Implants, 41-C Badar Commercial Street 10, Phase 5, Badar Commercial DHA, Karachi, Pakistan; 5 Department of Research, School of Public Health, Dow University of Health Sciences, Karachi, Pakistan

**Keywords:** Early childhood caries, Human beta defensin-3, Anemia

## Abstract

**Background:**

Human beta defensin-3 (HβD-3) is an antimicrobial peptide present in saliva that protects tooth surfaces from microbial attack. These peptides are part of innate immunity so levels may be affected by different systemic diseases like anemia. Therefore, anemia may predispose an affected child to an increased risk of dental caries. The objectives of this study were to determine the association of early childhood caries (ECC) with HβD-3 levels and observe the association of HβD-3 levels with childhood anemia.

**Methods:**

A total of 80 children admitted in a pediatric medical ward, age 48–71 months, of either sex were included in the study. The included children were categorized as cases (children with ECC n = 40) and controls (children without ECC n = 40). Children were further segregated into the anemic and non-anemic sub-groups based on the hospital record of hemoglobin level. The salivary concentration of HβD-3 was measured by Enzyme-Linked Immuno-sorbent assay (ELISA). IBM SPSS version 20 software was used for statistical analysis. Two sample t-test and one-way ANOVA were used to compare mean values while spearman was used for correlations at *p* < 0.05.

**Results:**

The mean Salivary HβD-3 level in cases (8.87 ± 4.30) was significantly higher (*p* = 0.042) as compared to controls (7.23 ± 2.57). Salivary HβD-3 level in patients with caries and without anemia was highest (10.80 ± 4.50) whereas salivary HβD-3 level in the presence of caries and anemia was lowest (6.94 ± 3.13) amongst all groups. This difference was statistically significant (*p* = 0.001). Salivary HβD-3 level was found to be moderately correlated with cases (*p* = 0.002). An inverse correlation was found between salivary HβD-3 level and anemia (r = -0.479, *p* = 0.002).

**Conclusion:**

Anemia may affect the innate immunity of children, and may result in a decreased level of salivary HβD3, thus increasing vulnerability to decay.

## Background

Dental caries in pre-school children commonly known as early childhood caries (ECC) remains the most prevalent chronic disease in children which has a significant impact on society [1]. ECC was found to be five times as common as asthma and seven times as frequent as hay fever when it was compared with other childhood diseases [2]. Early childhood caries has multifactorial etiology. Cariogenic microorganisms (mutans streptococci and lactobacillus) present in dental plaque are one of the main etiological factors [3]. Despite the role of diet and oral hygiene in its etiology, innate immunity may also play its part in caries development [4]. Previous publications have associated innate immunity markers such as lysozymes, lactoferrin [5], immunoglobulins [6], and antimicrobial peptides [7–9] with ECC [10].

Antimicrobial peptides (AMPs) are a part of the innate immune response that are present in the oral cavity and are important contributors in maintaining the balance between disease and health in the oral cavity. These include beta-defensins that are expressed by oral epithelium, alpha-defensins, secreted by neutrophils, or cathelicidin [10]. They have broad-spectrum antimicrobial activity against gram-positive and negative bacteria, some yeasts, and viruses and are being widely used as potential therapeutic agents especially in the oral cavity where there is a constant exposure of microorganisms [10]. AMPs prevent caries development by inhibiting the growth and adhesion of microorganisms or by inactivating its toxins [11]. These peptides are classified on their structural and biochemical basis. Defensin is a type of AMP with three disulfide bonds [12]. Among other peptides of this group, HβD-3 is important because of its structural and functional diversity and its recent coverage in pharmaceutical applications [13]. This peptide is either expressed constitutively or is inducible, and it contributes to innate immunity by direct bactericidal activity and adaptive immunity through effector and regulatory functions [13]. An Asian study in 2017 reported increased HβD-3 levels in saliva of carious group (6–10 year school children) as compared to healthy controls [8]. Similarly, increased salivary HβD-2 and histatin 5 have also been associated with an increased caries experience [9].

Children with a severe form of caries are malnourished due to poor feeding habits or food intake with disturbed sleep patterns due to constant pain [14]. Children experiencing pulpal involvement in at least one tooth can weigh less than those without it [15] and resulting malnutrition may leave a major impact on overall systemic health [16]. If such cases are not treated early, prolonged malnutrition not only makes the child anemic but may also affect salivary gland function by reducing its flow, constituents, and buffering capacity, thus, making a child more prone to decay [14–17]. Anemia is a common public health problem in growing children and there is a strong association between Iron Deficiency Anemia (IDA) and ECC [17, 18]. Iron deficiency anemia is suggested as the most common nutritional deficiency affecting almost 2 billion people in the world [19]^.^

Many theories have been put forward to explain the above-mentioned relationship. For example, the production of cytokines due to the enhanced inflammatory response of the body in ECC impairs erythropoiesis or prolonged episodes of pain that causes difficulty in chewing /eating and also results in malnutrition which eventually leads to low levels of Hb & IDA [20]. Childhood anemia also increases the susceptibility of children towards infection by its effects on defective Interleukin-2 (IL-2) and Interleukin-6 (IL-6) production and thus compromises immunity [21]. Iron has been categorized as an important component of the immune system as its deficiency decreases the bactericidal effects of innate host peptides [22]. Previous researches have reported that early childhood caries may have a direct relationship with HβD peptide [8, 9]. However, an inverse relationship, i.e. children with anemia are perhaps more prone to develop dental caries, may also be true. More specifically, can anemia alter the level of HβD-3 in children in the presence of dental caries? This is because innate immune response may be affected by a systemic disease like anemia? These questions were explored in the present study as many studies have been compiled in a systemic review that has discussed this relationship [18].

Considering two strong associations of ECC with Anemia and ECC with HβD-3, the purpose of this study was to observe the association of HβD-3 with ECC considering anemia as a risk factor.

## Methods

### Study design and setting

This case–control study was conducted in the Pediatric Ward of Ruth K.M.Pfau Civil Hospital Karachi after taking the research approval from the Institutional Review Board (Ref: IRB-1200/DUHS/Approval/2019/).

### Sample size

The sample size of five subjects per group was calculated using PASS version 11 software, based on two independent sample t-test allowing unequal variances with 95% confidence interval and 80% power of the test. Mean ± standard deviation of human beta-defensin in cases (with caries) 2.29 ± 0.05 and in controls (without caries) 2.15 ± 0.07 respectively [9]. Keeping in view low subjects in each group, we increased the sample size to 80 subjects (40 per group).

### Study participants

Both children, with and without caries, were recruited from the Pediatric Ward of Ruth K.M.Pfau Civil Hospital Karachi. Cases included children reporting with ECC in primary dentition with one or more decayed, missing, or filled teeth in any primary tooth in a child of either sex with age between 48 and 71 months. The controls included children without decayed teeth in primary dentition, of either sex of the same age group (48–71 months). However, un-cooperative children, differently-abled children, children older than this age group, or children with systemic disease other than anemia were not included.

The study population comprised 80 children, 40 children were with caries (ECC) as cases and 40 were without caries (ECC) as controls. Further stratification in cases and controls was performed based on hemoglobin level (taken from their previous medical records) with the reference value [23] (< 11.0 g/dl) into anemic and (> 11.0 g/dl) non-anemic groups during statistical analysis. These children, cases, and controls were sub-divided into the following four groups. Cases: Group I (ECC + Anemia+), Group II (ECC + Anemia−). Controls: Group III (ECC- Anemia+) and Group IV (ECC- Anemia−).

Consent was taken from parents at the time of recruitment of children in the study. Demographic details like age and sex with socio-economic data were collected and clinical examination was performed with examination instruments for detection of decayed, missing, and filled tooth index (dmft). Decayed, missing, and filled teeth were counted with related history. The findings were recorded in datasheets. Children were asked not to drink or eat for 30 min before sample collection.

### Saliva collection and quantitative assessment by ELISA

Unstimulated whole saliva was collected by asking children to sit upright, tilting their heads down, and keeping their mouths open until saliva flooded on the floor of the mouth. Then they were asked to let saliva drool into the falcon tube of 15 ml. The procedure was repeated until the required amount of saliva was collected (4-5 ml) [24]. Falcon tubes were kept in crushed ice in disposable glasses. The saliva sample was kept in crushed ice in thermo-pole bags, and in 30 min was shifted to the lab for centrifugation. All samples were centrifuged to clear saliva at 4500 rpm at 4 °C for 15 min. The supernatant was collected by using a micropipette. A 0.3 ml was collected into Eppendorf tubes and stored in the freezer (− 20 °C) until further analysis [22, 24, 25]. All the procedures were performed under relevant guidelines [26, 27].

The level of HβD-3 in saliva was estimated by using an Enzyme-Linked Immuno-sorbent assay (ELISA) kit (Human Beta Defensin 3 ELISA kit Cat. No E3240Hu) from Bioassay Technology Laboratory.

### Statistical analysis

IBM SPSS version 20 software was used to analyze data. Statistical analysis was conducted based on data distribution by using two independent samples t-test to compare the mean HβD-3 level in cases and controls. One way-ANOVA was used to compare mean values of four stratified groups of cases and controls. A post hoc test (Tukey’s test) was used for the pairwise comparison of group II (children with caries and without anemia) with the remaining three groups. Mann–Whitney test was used to find the difference in the dmft index in different study groups of ECC and anemia. Spearman correlation was used for observing the correlation between HβD-3 with ECC & HβD-3 with anemia.

## Results

Basic demographic statistics are presented in Table 1, which shows the distribution of the population by age and sex into different study groups. The age group included children from 48–71 months with a mean value of 65.0 ± 4.4. The mean salivary HβD-3 level in cases (8.87 ± 4.30) was significantly higher (*p* = 0.042) as compared to controls (7.23 ± 2.57) as presented in Table 2. When cases and controls were further stratified into four groups, salivary HβD-3 level in the presence of caries and anemia (group I) was the lowest (6.94 ± 3.13, *p* = 0.001) and was highest 10.80(4.50) in children with caries and without anemia (group II) amongst all groups as presented in Table 3. The pairwise analysis of groups further strengthened this relationship and revealed that the mean HβD-3 level of group II was highest among all groups.

**Table 1:** Descriptive statistics of sex, age and Hb

Characteristics	n = 80 (%)	Anemic: non-anemic (ratio)	ECC: non-ECC (ratio)	Cases (with ECC) n = 40 (%)	Controls (without ECC) n = 40 (%)
*Sex*					
Female	47 (58.7)	22:25	18:29	29 (72.5%)	18 (45%)
Male	33 (41.25)	18:15	22:11	11 (27.5%)	22(55%)
Age (months)^a^	65.0 ± 4.4				
Hb level^a^ (g/dl)	11.6 ± 1.6				

Hb, Hemoglobin; ECC, Early childhood caries

^a^Values are represented as Mean ± SD

**Table 2:** Salivary HBD-3 levels in different study groups

Study groups	n = 80 (%)	HBD-3 levels Mean ± SD	t-test (*p*-value)^a^
*ECC*			
Cases (with ECC)	40 (50)	8.87 ± 4.30	2.064 (0.042)^a^
Controls (without ECC)	40(50)	7.23 ± 2.57	
*Anemia*			
With anemia	40(50)	7.15 ± 2.82	− 2.482(0.015)^a^
Without anemia	40(50)	9.02 ± 3.96	
*Age groups*			
48–59 Months	22(27.5)	8.31 ± 3.67	0.730^b^
60–71 Months	58(72.5)	7.95 ± 3.62	
*Gender*			
Males	33(41.25)	7.54 ± 2.67	0.292^a^
Females	47(58.75)	8.41 ± 4.14	

^a^t-test

^b^Mann whitney test

**Table 3:** Salivary HβD-3 levels among different study groups

Groups	Mean (SD)	95% C.I	*P*-value^b^
I (children with ECC and Anemia)	6.94 (3.13)	5.4–8.4	0.001^b^
II (children with ECC but without Anemia)	10.80 (4.50)	8.7–12.9	
III (children without ECC but with Anemia)	7.22 (2.87)	5.8–8.5	
IV (children without ECC and Anemia)	7.25 (2.30)	6.1–8.3	
*Comparison of group II(children with ECC and without Anemia) with all other groups by Tukey/HSD test*			
Mean difference			
Group I (children with ECC and Anemia)	3.866^a^	0.002^c^	
Group III (children without ECC but with Anemia)	3.579 ^a^	0.005^c^	
Group IV (children without ECC and Anemia)	3.579 ^a^	0.006^c^	

SD, standard deviation; CI, confidence interval

^a^Mean difference

^b^One way anova

^c^Post Hoc Tukey test

The association of dmft with ECC and anemia is presented in Table 4. The dmft score of anemic children among cases (group I) was higher (6.20 ± 1.73) as compared to non-anemic children (group II). This difference was statistically significant 4.257 (0.000) (Table 4).

**Table 4:** Dmft index in different study groups

Study groups	Dmft Median (IQR)	*p*-value^a^
*ECC*		
Carious	5.0 (4.0–6.8)^b^	< 0.001^a^
Non-carious	0.5 (0–1.0)^b^	
*Anemia*		
Anemic	2.5 (1.0–6.75)^b^	0.134^a^
Non-anemic	2.5 (0.0–4.0)^b^	

Cases (with ECC)	Mean ± SD	t-test (*p*-value^c^)
*Dmft score for the children with and without anemia in children with caries*		
Group I (children with ECC and Anemia)	6.20 ± 1.73	4.257(0.000)^c^
Group II (children with ECC without Anemia)	4.35 ± 0.88	

^a^Mann-Whitney

^b^Median (inter quartile range); Dmft (decayed, missing and filled tooth), SD (standard deviation)

^c^t-test

The level of salivary HβD-3 and anemia between cases and control was found to be negatively correlated with cases (r = − 0.479, *p* = 0.002) (Table 5). An inverse correlation was found between salivary HβD-3 level & anemia (r = − 0.262, *p* = 0.019), which explains the role of anemia in affecting innate immunity peptides (Table 5). When HβD-3 was correlated with ECC separately, a weak correlation was found (r = 0.12, *p* = 0.294).

**Table 5:** Spearman correlation(r) of HβD-3 with different study groups

HβD-3 with	R (*P*-value)^a^
Cases	− 0.479 (0.002)^a^
Controls	0.017 (0.919)^a^
Anemia	− 0.262 (0.019)^a^

^a^Spearman correlation

## Discussion

This research is the first original research to observe the effects of anemia on salivary levels of HβD-3 in association with ECC. Our results suggest that although the presence of caries may increase the salivary levels of HβD-3, the concomitant presence of anemia with caries may actually cause a reduction in these protective peptides. This research presented a correlation between ECC, HβD-3, and anemia when all three variables were compared together. The results showed high levels of peptides in children with ECC (cases) as compared to children without ECC (controls). Upon further stratification (on basis of anemia) of cases & controls into four subgroups, it was found that group II from cases had the highest levels of peptides as compared to all other groups. Whereas, the levels of peptides in group I were the lowest as compared to the other three groups demonstrating that being anemic reduces the levels of peptides.

The present research showed that cases had higher levels of HβD-3 as compared to controls, which agrees with previous research findings of Indrawati et al. and Jurczak et al. [8, 9]. However, other peptides have also been associated with ECC in past with contrasting results. Indrawati reported a greater expression of HβD-1 in caries-affected children when all three beta-defensins were compared [8]. Jurczak et al. discovered increased levels of HβD-2 and histatin 5. Joly et al. used radial diffusion to test the antimicrobial activity of HβD-2 and HβD-3 against a variety of oral micro-organisms and recognized HβD-3 with higher antimicrobial activity as compared to HβD-2 [28]. While Davidopouloou et al. [7] & Malcolm et al. found high levels of cathelicidin LL-37 [29]. Renulka et al. described the role of the β-defensins gene, which is connected to both low and high-risk caries [30]. Defensins are a part of a small arginine-rich peptide family, and arginine in its free or associated form shows strong protective property against caries [31]. Hence, increased levels of peptides are associated with increase caries incidence.

Our findings show that the dmft index of anemic children (6.20 ± 1.73) was higher as compared to non-anemic children (4.35 ± 0.88) (Table 4), which may be attributed mainly to the dietary patterns and other factors affecting the overall health of these children. These results were in agreement with previous findings [32].

Our study is perhaps the first to concurrently observe the effects of anemia on salivary levels of HβD-3 in association with ECC. The two-way relationship between anemia & ECC was previously supported by many researchers. Due to a reduced amount of red blood cells or low hemoglobin levels at a suboptimal level, cells or tissues are unable to maintain normal physiological function that relies on micronutrients [33]. Hence anemia not only affects the physical and mental growth of the child but also affects the defense system of the oral cavity. It weakens the immunity of a child to fight against microorganisms making the child more prone to infections and decay [19]. Previous researches have addressed this significant inverse correlation between anemia and caries incidence [19]. Bansal et al. and Koppal et al. reported that children with lower hemoglobin levels have a high incidence of caries [17]. Abdallah et al. also associated a higher dmft index with decreasing hemoglobin level [32]. Babu et al. also found an inverse significant relationship between serum hemoglobin level and ECC [34]. Tang et al. found 46% of children suffering from Iron deficiency anemia (IDA) in their research on ECC [35]. Shaoul et al. also found a strong correlation between ECC and IDA, their results showed that treating carious lesions on time improves iron deficiency in children [36]. Another research concluded that children with severe early childhood caries appear to have significantly lower hemoglobin levels with greater odds of developing IDA as compared to caries-free children [32]. Anemia occurs commonly because of inadequate feeding practices, micronutrient deficiency, and frequent infections [37]. IDA is most common in developing countries, which affects nonspecific immunity in many ways. Iron is important for proper cell differentiation and growth. It is an essential component of the proper enzymatic functions of immune cells. It is needed for regulation in cytokine production and the mechanism of the second messenger system. Hence, its deficiency is considered important in molecular and cellular defects responsible for immune deficiency [38].

## Strengths and limitations

We have determined the relationship of salivary levels of HβD-3 in cases and controls based on our study design. We have shown that cases (children with ECC) have higher levels of the peptide as compared to controls (children without ECC). But can this approach be useful in assessing the risk of caries in children as a predictive tool and can it prevent the progression of the disease? These questions can be the basis of future research. This research, however, has proposed the role of anemia in children as anemic children with caries had the lowest level of peptides.

There may be some limitations in this study, the present research had a small sample size, and, therefore, to extrapolate the results to the general public and to truly realize the potential of this peptide as a diagnostic tool, studies with a larger sample size may be performed. Parental education and attitude also influence the feeding practices and oral hygiene habits of children. Apart from salivary immune components, these factors are also very important for the propagation of caries because ECC is a multifactorial disease and is influenced by multiple factors including social and behavioral factors. The present research lacks data on nutrition, their feeding and oral hygiene habits, and the influence of parents in all study groups. Selection bias is one of a major weakness of case–control study design due to lack of random sampling however was partially addressed by selecting the controls from the same pool of a population from where the cases were selected as almost all patients in the present research were from the low socioeconomic strata as all the samples were collected from government sector hospitals.

## Conclusions

The following conclusions may be drawn within the limitations of this study:

The HβD-3 level increased with increasing caries incidence. Children experiencing anemia and ECC concomitantly had the lowest level of peptides showing that anemia affects innate immunity, which may increase vulnerability to decay in children under the age of 71 months. However, HβD-3 alone cannot be considered as a biomarker to measure the risk of caries as ECC and anemia both are multifactorial diseases.

## Data Availability

The datasets used and/or analyzed during the current study are available from the corresponding author on reasonable request.

## Abbreviations

ECC: Early childhood caries
Hb: Hemoglobin
IDA: Iron deficiency anemia
HβD: Human beta defensins
AMPs: Anti-microbial peptides
WHO: World Health Organization
DMFT: Decayed missing filled teeth
IL: Interleukin
